# MLST-Based Analysis and Antimicrobial Resistance of *Staphylococcus epidermidis* from Cases of Sheep Mastitis in Greece

**DOI:** 10.3390/biology10030170

**Published:** 2021-02-24

**Authors:** Eleni I. Katsarou, Dimitris C. Chatzopoulos, Themis Giannoulis, Katerina S. Ioannidi, Angeliki I. Katsafadou, Panagiota I. Kontou, Daphne T. Lianou, Zissis Mamuris, Vasia S. Mavrogianni, Charalambia K. Michael, Elias Papadopoulos, Efthymia Petinaki, Styliani Sarrou, Natalia G. C. Vasileiou, George C. Fthenakis

**Affiliations:** 1 Veterinary Faculty, University of Thessaly, 43100 Karditsa, Greece; elekatsarou@vet.uth.gr (E.I.K.); vetdchatzop@gmail.com (D.C.C.); kate_ioan@windowslive.com (K.S.I.); agkatsaf@vet.uth.gr (A.I.K.); dlianou@vet.uth.gr (D.T.L.); vmavrog@vet.uth.gr (V.S.M.); cmichail@vet.uth.gr (C.K.M.); 2 Faculty of Animal Science, University of Thessaly, 41110 Larissa, Greece; themisgia@gmail.com (T.G.); vasileiounat@gmail.com (N.G.C.V.); 3 Department of Computer Science and Biomedical Informatics, University of Thessaly, 35131 Lamia, Greece; pankontou@gmail.com; 4 Faculty of Biochemistry and Biotechnology, University of Thessaly, 41110 Larissa, Greece; zmamur@bio.uth.gr; 5 Laboratory of Parasitology and Parasitic Diseases, School of Veterinary Medicine, Faculty of Health Sciences, Aristotle University of Thessaloniki, 54124 Thessaloniki, Greece; eliaspap@vet.auth.gr; 6 University Hospital of Larissa, 41110 Larissa, Greece; petinaki@uth.gr (E.P.); stsarrou@yahoo.gr (S.S.)

**Keywords:** antibiotic resistance, genetic diversity, intramammary infection, mastitis, meta-analysis, ovine, sheep, spatiotemporal spread, *Staphylococcus*

## Abstract

**Simple Summary:**

Data regarding *Staphylococcus epidermidis* in the multi-locus sequence typing (MLST) database are appraised. Further, work on the association with the resistance of *Staphylococcus epidermidis*, isolated from cases of sheep mastitis in Greece, to antibiotics is presented. The database includes 1593 isolates from 46 countries, most (76%) of human origin. Of the isolates of animal origin, 72% are from cases of mastitis, among which there were differences between isolates of bovine or ovine origin. Of the isolates from sheep mastitis in Greece, in 58%, resistance to antibiotics was found. There was no association between MLST types and resistance to antibiotics, but antibiotic resistance was more frequent among isolates from flocks being hand- than machine-milked.

**Abstract:**

*Staphylococcus epidermidis* is an important causal agent of ovine mastitis. A literature search indicated a lack of systematic studies of causal agents of the infection by using multi-locus sequence typing (MLST). The objectives were to analyse MLST-based data and evaluate the antimicrobial resistance of *S. epidermidis* isolates from ovine mastitis in Greece. The database included 1593 isolates from 46 countries: 1215 of human, 195 of environmental and 134 of animal origin, distributed into 949 sequence types (STs) and cumulatively with 450 alleles therein. Among mastitis isolates, bovine isolates were distributed into 36 different STs and ovine ones into 15 STs. The 33 isolates from ovine mastitis in Greece were in 15 different STs, 6 of these (ST677, ST678, ST700, ST 709, ST710, ST711) assigned for the first time; in addition, 5 alleles (65 for *arcC*, 59 for *aroE*, 56 and 57 for *gtr* and 48 for *tpiA*) were identified for the first time. The spanning tree of these isolates included 15 nodes and 14 edges (i.e., branches). Among these isolates, 19 showed resistance to antimicrobial agents (tetracycline, penicillin, fucidic adic, erythromycin, clindamycin, cefoxitin). Resistance-related genes (*tetK*, *tetT*, *msrA*, *tetM*, *tetS*, *ermC*, *mecA*) were detected. There was no association between STs and resistance to antimicrobial agents. Isolates with antimicrobial resistance were recovered more often from flocks where hand-milking was practised.

## 1. Introduction

Methods for bacterial identification and evaluation have evolved rapidly in recent years. One such method is multi-locus sequence typing (MLST), which is a tool for understanding the dynamics of pathogens and for gaining insights into their genetic diversity. The method is based on the typing of multiple loci, specifically by characterising bacterial isolates using the deoxyribonucleic acid (DNA) sequences of internal fragments of multiple housekeeping genes. For each of these genes, the various sequences in a bacterial species are assigned as distinct alleles; then, the alleles at each of the loci define the allelic profile of the isolate, termed sequence type (ST) [1,2,3].

For staphylococci, MLST is available for Staphylococcus aureus, Staphylococcus chromogenes, Staphylococcus epidermidis, Staphylococcus haemolyticus, Staphylococcus hominis and Staphylococcus pseudointermedius. Specifically for S. epidermidis, the seven genes used in the typing scheme are arcC, aroE, gtr, mutS, pyrR, tpiA and yqiL.

*S. epidermidis* is well adapted to humans and may be present in large populations as part of the skin microflora of people [4,5,6]. Indeed, Kloos [4] regarded this species as predominantly human.

In Greece, *S. epidermidis* has been reported as a primary cause of bacteraemia in adults, following intravenous catheterisation [7,8], transplantations [9] or the use of prosthetic devices [10], as well as in neonates [11,12,13]. It has also been reported as an important cause of endocarditis [14]. The organism was also isolated from contaminated soft lenses [15], as well as from the eyes of hospitalised patients [16].

With regard to animal infections, *S. epidermidis* has been repeatedly recognised as an important causal agent of mastitis. Holmberg [17] was the first to detail the importance of *S. epidermidis* as a mammary pathogen. Thereafter, this was confirmed by many other researchers. In addition, studies performed in sheep have indicated the importance of the organism as a mastitis pathogen in that animal species [18,19,20].

The organism was repeatedly recovered from food of animal origin, specifically cured meat products [21], fish [22] and fish products [23]; in all cases, it was the most frequently identified coagulase-negative species.

With regard to mastitis in sheep, in Italy, Turchi et al. [24] reported *S. epidermidis* as the predominant coagulase-negative staphylococcal species from cases of the infection in sheep. In Greece, Kalogridou-Vasileiadou [25], Fthenakis [26] and Kiossis et al. [27] reported that it was the most frequently recovered coagulase-negative species from cases of ovine mastitis: 19%, 56% and 60%, respectively, among those bacteria.

Ovine mastitis is an important infection, which leads to significantly reduced production in ewes and adversely affects their welfare standards. The European Food Safety Authority [28] has recognised mastitis as the most important disease for welfare concerns in ewes. Recently, Vasileiou et al. [29] performed an extensive investigation of mastitis in flocks in Greece and reported that the prevalence of mastitis was 26%. Mastitis leads to reduced production in affected ewes; Saratsis et al. [30] reported up to 55% reduction in milk yield, coupled with a simultaneous increase in somatic cell counts in milk, which results in penalties in the milk price.

Although *S. epidermidis* is an important causal agent of sheep mastitis, a recent detailed study of the international literature on ovine mastitis [31] indicated that no MLST-based studies on the causal agents of the infection have been reported. The objectives of this work were to analyse MLST-based data and evaluate the antimicrobial resistance patterns of *S. epidermidis* isolates from cases of ovine mastitis in Greece.

The manuscript is structured in two parts. In the first part, data publicly available in the MLST database were analysed and presented in detail; in the second part, the work performed on 33 *S. epidermidis* isolates recovered from ovine mastitis in Greece is presented. To the best of our knowledge, this is an original report on the detailed MLST-based evaluation of *S. epidermidis*, as well as an original application of MLST in ovine mastitis isolates.

## 2. Materials and Methods

### 2.1. Data Collection Regarding S. epidermidis from the MLST Database and Analysis

#### 2.1.1. Data Collection

The details of all *S. epidermidis* isolates, as available in the multi-locus sequence typing (MLST) database (www.pubmlst.org (accessed on 10 November 2020)), were downloaded and entered into Microsoft Excel and Access (Microsoft Corporation, Redmond, WA, USA) for calculations. The database was last accessed on 21 December 2020.

The isolates recovered from cases of mastitis in animals were assessed further. A spanning tree of the isolates was constructed using the Grape-Tree application [32], which is linked directly to the MLST database. The following parameters were employed for the assessment of all the mastitis isolates from animals: (a) selected loci: *arcC*, *aroE*, *gtr*, *mutS*, *pyrR*, *tpiA* and *yqiL*; (b) scheme: MLST; and (c) fields: according to the desired graphic output (e.g., isolate ID, ST).

#### 2.1.2. Statistical Analysis

Initially, descriptive analyses were performed with all the isolates in the database taken into account. Thereafter, analysis of the isolates from cases of mastitis was performed; the frequencies of alleles in each of the seven genes used for MLST were compared in tables of cross-categorised frequency data by using the Pearson chi-square test or the Fisher exact test, as appropriate. Statistical significance was considered at *p* < 0.05.

### 2.2. Evaluation of S. epidermidis Isolates Recovered in Greece

#### 2.2.1. Bacterial Isolation

A total of 33 *S. epidermidis* isolates, which had been recovered (2016–2018) during field investigations of mastitis in Greece, were evaluated in detail.

One of the investigations referred to a longitudinal study on the prevalence and aetiology of ovine mastitis in 111 sheep flocks in the 13 administrative regions of the country [29]. The second investigation referred to a cross-sectional trial performed in five flocks, in each of which nine visits were paid throughout a lactation period [33].

Among the 111 flocks in the longitudinal study, the management system applied was intensive in 26 (23.4%), semi-intensive in 57 (51.4%), semi-extensive in 22 (19.8%) and extensive in 6 (5.4%) flocks (management system according to the definitions of the European Food Safety Authority [28]); machine-milking was performed in 74 (66.7%) and hand-milking in 37 (33.3%) flocks. Among the five flocks in the cross-sectional study, the management system applied was intensive in two (40.0%) and semi-intensive in three (60.0%); machine-milking was performed in three (60.0%) and hand-milking in two (40.0%) flocks.

Milk samples were collected from individual ewes for bacteriological and cytological examinations. Established bacteriological techniques were used for primary isolation, and provisional identification of the isolates as *Staphylococcus* spp. was performed. Then, isolates among them, selected at random, were further identified to the species level by using the Vitek^®^ 2 automated system (BioMerieux, Marcy-l’-Étoile, France). The identity of all the isolates under evaluation was confirmed to be *S. epidermidis*.

#### 2.2.2. MLST and Phylogenetic Analysis

Initially, the isolates were typed by using MLST. This was performed by following the relevant guidelines: https://pubmlst.org/organisms/staphylococcus-epidermidis (accessed on 10 November 2020) [34,35]. Details of primers used are in Table S1. By employing these primers, internal fragments of the seven genes were amplified by means of polymerase chain reaction (PCR) and chromosomal DNA of *S. epidermidis* isolates as a template. PCR involved initial denaturation at 95 °C for 3 min; this was followed by 34 cycles at 95 °C for 30 s, 50 °C for 1 min and 72 °C for 1 min, with a final extension at 72 °C for 10 min.

The PCR products of the seven genes were purified with the PureLink™ PCR Purification Kit (Thermo-Fisher Scientific-Invitrogen, Waltham, MA, USA) and subsequently sequenced. Sanger sequencing was performed in order to identify the allelic profile of the *S. epidermidis* strains. The sequencing was performed in an ABI3730xl DNA analyser (CD-Genomics, New York, NY, USA). The exported data were analysed by using MEGA software (Center for Evolutionary Medicine and Informatics, Tempe, AZ, USA) and BLAST (National Center for Biotechnology Information, Bethesda, MD, USA). The details of the isolates were entered into the database.

A spanning tree of the isolates was constructed, as detailed above (Section 2.1.1). For phylogenetic analysis, the allelic sequences were retrieved and concatenated in one contig. For the missing alleles, an imputation method was used based on the available haplotype information: by using the option *Search or Browse profiles* (https://pubmlst.org/bigsdb?db=pubmlst_sepidermidis_seqdef&page=query&scheme_id=1 (accessed on 10 November 2020)), the successfully genotyped alleles were used as a query to retrieve the most probable haplotype, according to the deposited haplotypes.

In the majority of cases, the combinations of retrieved alleles were indicative of one single record, which was used as the most probable ST. The haplotype sequence of *S. aureus* ST1 was retrieved to be used as an outgroup in the analysis. The concatenated sequences were aligned using MUSCLE [36], and the alignments were refined by eye. The appropriate substitution model was detected using jModelTest 2 [37], and the phylogenetic tree was constructed using a maximum likelihood (ML) method with 100 bootstrap replications in MEGA X [38].

#### 2.2.3. Susceptibility Testing to Antimicrobial Agents

Susceptibility testing to 11 antimicrobial agents (ampicillin, azithromycin, cefoxitin, clarithromycin, clindamycin, erythromycin, fosfomycin, fucidic acid, penicillin, tetracycline and trimethoprime-sulfamethoxazole) was carried out by the Vitek^®^ 2 automated system.

The isolates, regardless of their phenotype, were tested by PCR for the following genes: *ermA*, *ermB*, *ermC*, *ermT*, *ermY*, *lnuA*, *lnuB*, *lnuC*, *lsaA*, *mecA*, *mphC*, *msrA*, *tetK*, *tetL*, *tetM*, *tetS*, *tetT* and *vgaA*. For DNA template preparation, 1 mL of a bacterial cell suspension to a final turbidity equivalent to 1.0 Mc Farland standard was centrifuged at 13,000 rpm for 10 min. The pellet was re-suspended in 0.1 mL of lysis buffer (50 mM Tris-HCl (pH 7.5), 1% Triton X-100, 1 mM ethylenediaminetetraacetic acid (EDTA) and 200 mg mL^−1^ of proteinase K) The mixture was incubated for 3 h at 37 °C and then boiled (100 °C) for 10 min and clarified by centrifugation [39]. Details of the primers and conditions employed are in Table S2. Amplification was performed in a Veriti 9902 Thermocycler (Applied Biosystems, Foster City, USA). *S. epidermidis* American Type Culture Collection (ATCC) 12228^TM^ (American Type Culture Collection, Manassas, VI, USA) was the quality control organism in the Vitek^®^ 2 automated system.

#### 2.2.4. Data Management and Analysis

Subclinical mastitis was defined in ewes with no clinical mammary signs, in which a bacteriologically positive milk sample ((a) >10 colonies of the same organism and (b) no more than two different types of colonies) with concurrently increased leucocyte numbers therein (as detected by the California Mastitis Test (score ≥ l)) plus increased neutrophil and lymphocyte proportions (≥65% of all leukocytes) [40] was found. Mammary carriage was considered in ewes in which a bacteriologically positive milk sample with no increased CMT score (≤trace) or neutrophil and lymphocyte proportion (<65% of all leukocytes) was detected [29].

After susceptibility testing, interpretation of the results was based on criteria of the European Committee on Antimicrobial Susceptibility Testing (EUCAST) (http://www.eucast.org (accessed on 10 November 2020)) (more recent access date: 15 November 2020). Based on susceptibility testing results, isolates were classified as resistant or susceptible to each antimicrobial agent according to EUCAST criteria; no intermediate isolates were detected during the evaluation, and this possible result was omitted from analyses. Multi-drug resistant (MDR) isolates were those found resistant to at least three different classes of antimicrobial agents [41].

Evaluations of associations were performed in tables of cross-categorised frequency data by using the Pearson chi-square test or the Fisher exact test, as appropriate. The frequency of resistance to antimicrobial agents was evaluated against the various husbandry factors practiced in the farm where the isolates were recovered. The following husbandry practices were included: management system applied in the flock, stage of the lactation period at sampling, milking technique applied in the flock, application of post-milking teat-dipping, intramammary administration of antimicrobial agents at the end of the lactation period and vaccination against staphylococcal mastitis (Table S3). These details had been obtained through interviews with the respective farmers at the time of each visit.

A multivariable analysis was applied. Variables were removed from the initial model by backwards elimination. The *p*-value of removal of a variable was assessed by the likelihood ratio test, and for those with a *p*-value of >0.2, the variable with the largest probability was removed. This process was repeated until no variable could be removed with a *p*-value of >0.2. The final multivariable test required the following variables: (a) management system applied in the flock, (b) milking technique in the flock and (c) vaccination against staphylococcal mastitis.

Statistical significance was considered at *p* < 0.05.

## 3. Results

### 3.1. Details of S. epidermidis in the MLST Database

#### 3.1.1. Details of All *S. epidermidis* Isolates in the Database

In total, the database included 1593 isolates. Of these, 1215 (76.3%) isolates were of human origin, 195 (12.2%) isolates were of environmental origin and 134 (8.4%) isolates were of animal origin; for the remaining 49 (3.1%) isolates, no source was indicated. Among the animal isolates, most (*n* = 97, 72.4%) were recovered from cases of mastitis.

**Figure 1:**
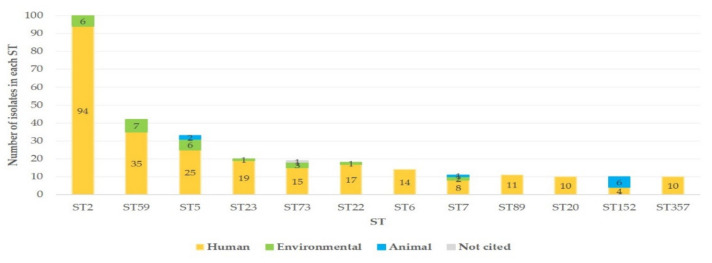
Histogram of the number of *Staphylococcus epidermidis* isolates in STs with ≥10 isolates within each ST, in the MLST database, according to the number of isolates distributed into each one and the source of the isolates (depicted in the colour of the bars). MLST: multi-locus sequence typing; ST: sequence type.

These isolates were distributed into 949 different STs. The STs with most isolates were ST2 (*n* = 100 isolates, 6.3%), ST59 (*n* = 42, 2.6%) and ST5 (*n* = 33, 2.1%), with, in total, 12 STs (1.3%) with ≥10 isolates (Figure 1). Most STs (*n* = 784, 82.6%) had one isolate.

**Figure 2:**
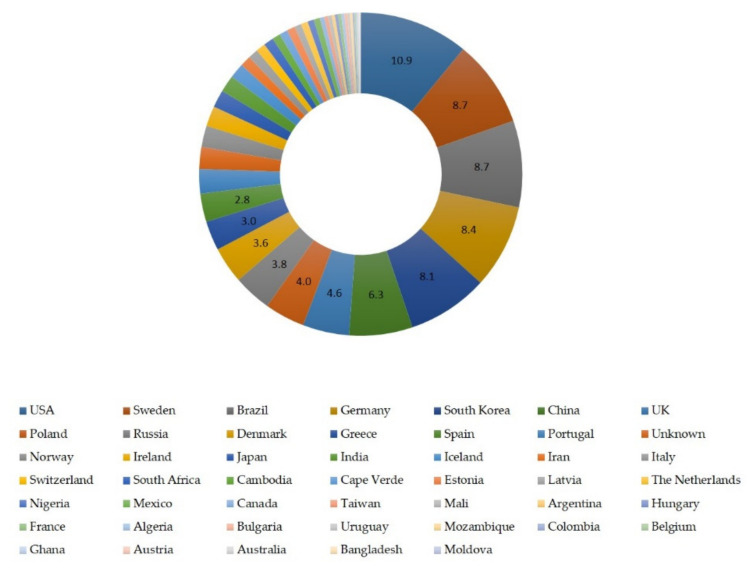
Ring pie chart of the frequency of *S. epidermidis* isolates in the MLST database (*n* = 1593) according to the country of origin. MLST: multi-locus sequence typing.

Geographically, isolates originated from 46 countries worldwide. Most isolates originated from the United States (*n* = 174, 10.9%), Sweden (*n* = 139, 8.7%), Brazil (*n* = 138, 8.7%), Germany (*n* = 134, 8.5%), South Korea (*n* = 129, 8.1%) and China (*n* = 101, 6.3%) (Figure 2).

Chronologically, 269 (16.9%) isolates were recovered up to 2000, 630 (39.5%) isolates were recovered from 2001 to 2010 and 539 (33.8%) isolates were recovered from 2011 to 2020. For 155 (9.7%) isolates, no year of recovery was indicated.

Cumulatively and across all 7 genes, 450 different alleles were detected in the 1593 isolates (Table 1).

#### 3.1.2. *S. epidermidis* Isolates Recovered from Mastitis

**Figure 3:**
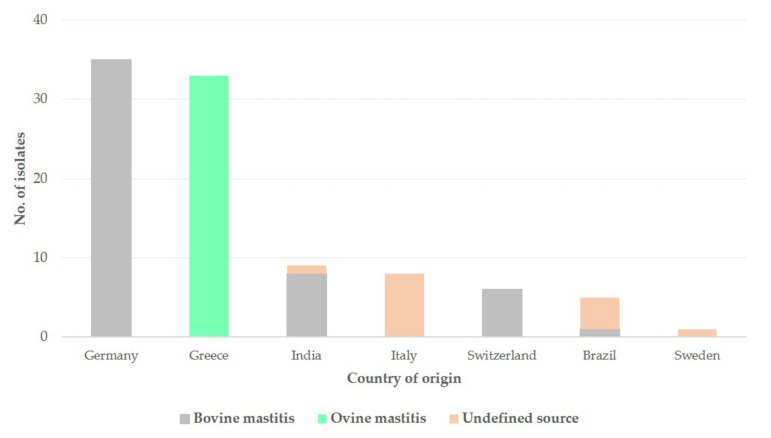
Histogram of the frequency of *S. epidermidis* isolates from cases of mastitis in the MLST database (*n* = 97) according to the country of origin and the source (cattle, sheep). MLST: multi-locus sequence typing.

The 97 *S. epidermidis* isolates included in the database, which were recovered from mastitis (*n* = 50 from cattle, *n* = 33 from sheep, *n* = 14 with no description of source (Figure 3)), were distributed to 59 different STs, while 1 isolate was untyped. The STs with most isolates were ST100 (*n* = 8 isolates, 8.2%), ST59 (*n* = 7, 7.2%) and ST91 (*n* = 6, 6.3%).

Geographically, isolates originated from seven countries, as follows: Germany (*n* = 35, 36.1%), Greece (*n* = 33, 34.0%), India (*n* = 9, 9.3%), Italy (*n* = 8, 8.2%), Switzerland (*n* = 6, 6.2%), Brazil (*n*= 5, 5.2%) and Sweden (*n* = 1, 1.0%) (Figure 3).

Chronologically, 49 (50.5%) isolates were recovered from 2001 to 2010 and 48 (49.5%) isolates were recovered thereafter.

There was evidence of a difference in the frequency of alleles of the genes *arcC* (*p* = 0.002), *aroE* (*p* = 0.0005), *gtr* (*p* = 0.0005), *tpiA* (*p* = 0.0010) and *yqiL* (*p* = 0.0017) between isolates from cases of mastitis and carrier-state isolates from animals in the same countries; no such difference was seen in the frequency of alleles of the other two genes (*p* > 0.09).

The isolates from cattle were distributed into 36 different STs (more frequently, ST91 and ST59) and the ones from sheep into 15 STs (*p* = 0.008). When isolates from cattle or sheep were compared, there was evidence of a difference in the frequency of alleles of the genes *arcC* (*p* < 0.001) and *yqiL* (*p* = 0.001) (Table 2) but not in the frequency of alleles of the other five genes (*p* > 0.21).

**Figure 4:**
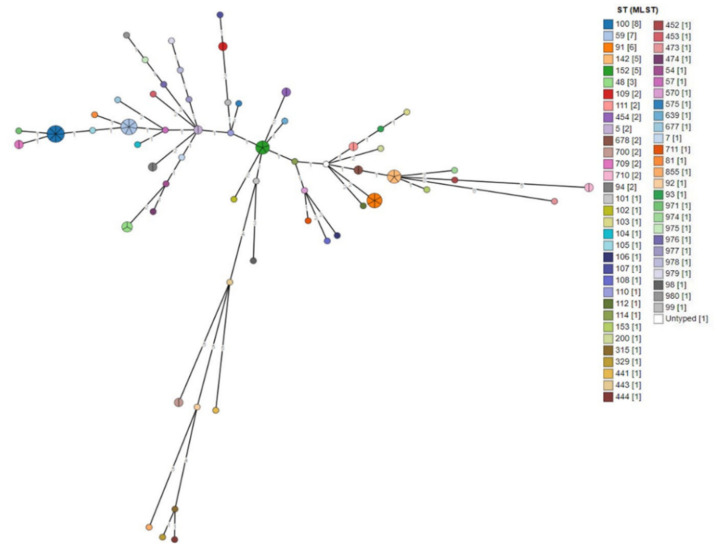
MLST-based spanning tree of *S. epidermidis* isolates from cases of mastitis in the MLST database (*n* = 97) according to the ST (drawn by using the Grape-Tree application). Each colour corresponds to an ST, the number in brackets corresponds to the number of isolates distributed into that ST, the colour of each node corresponds to the ST included in it and the number within the edges (i.e., branches) indicates the allelic distance. MLST: multi-locus sequence typing; ST: sequence type.

The spanning tree of all the isolates recovered from cases of mastitis is shown in Figure 4. The tree includes 60 nodes (which represent STs) and 59 edges (i.e., branches) (which mark allelic distance) (median length of edges: 1). In ST100, there were 8 isolates (7 isolates from sheep in Greece, 1 from cattle in Germany); in ST59, 7 isolates (5 isolates from cattle in Germany, 1 from cattle in Brazil, 1 from sheep in Greece); in ST91, 6 isolates (all from cattle in Germany); and in ST142 and ST152, 5 isolates each (all from sheep in Greece).

Of the 59 STs in which mastitis isolates were distributed, in 17 (28.8%), there were also isolates of human origin. There was no difference in the proportion of STs with isolates of bovine or ovine origin, and isolates of human origin were also distributed (27.8% versus 40.0%; *p* = 0.30).

### 3.2. S. epidermidis Isolates Recovered from Ovine Mastitis in Greece

#### 3.2.1. MLST

All the isolates from ovine mastitis (*n* = 33) in the MLST database, had been recovered in Greece from confirmed cases of subclinical mastitis. The allelic profiles of the isolates are in Table S4.

The 33 isolates were distributed into 15 different STs. The STs with most isolates were ST100 (*n* = 7 isolates, 21.2%) and ST142 and ST152 (*n* = 5 isolates each, 15.2%). In total, 6 STs were assigned for the first time with these isolates: ST677 (*n* = 1 isolate), ST678 (*n* = 1), ST700 (*n* = 2), ST 709 (*n* = 2), ST710 (*n* = 2) and ST711 (*n* = 1). Moreover, 5 alleles were also identified for the first time in these isolates: 65 for *arcC*, 59 for *aroE*, 56 and 57 for *gtr* and 48 for *tpiA*.

**Figure 5:**
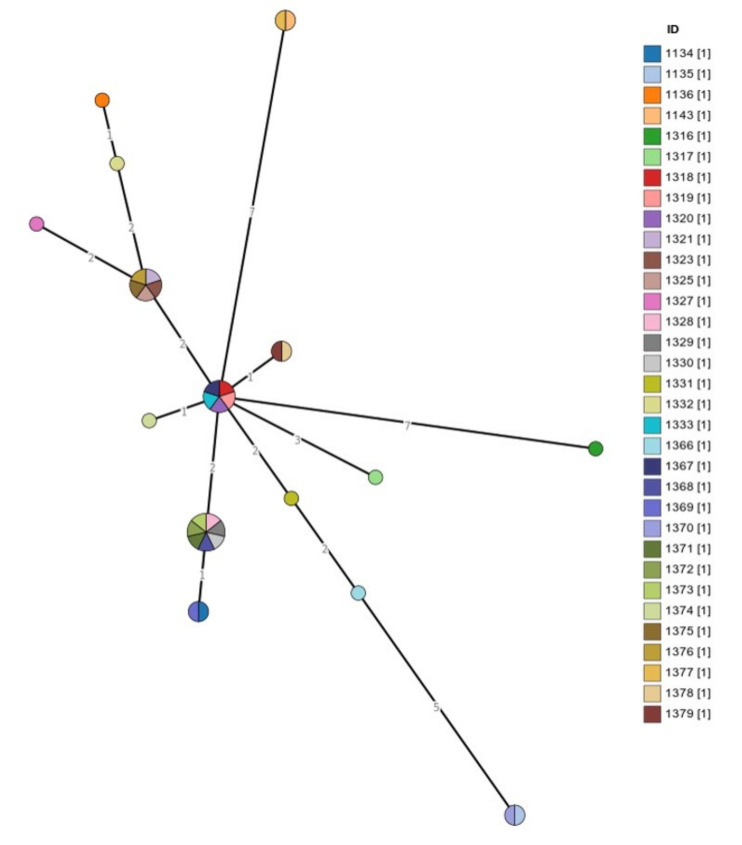
MLST-based spanning tree of 33 *S. epidermidis* isolates from cases of ovine mastitis in Greece in the MLST database according to isolate identity (drawn by using the Grape-Tree application). Each colour corresponds to an ST, the number in brackets corresponds to the number of isolates distributed into that ST, the colour of each node corresponds to the ST included in it and the number within the edges (i.e., branches) indicates the allelic distance. MLST: multi-locus sequence typing; ID: isolate identity in the MLST database.

**Figure 6:**
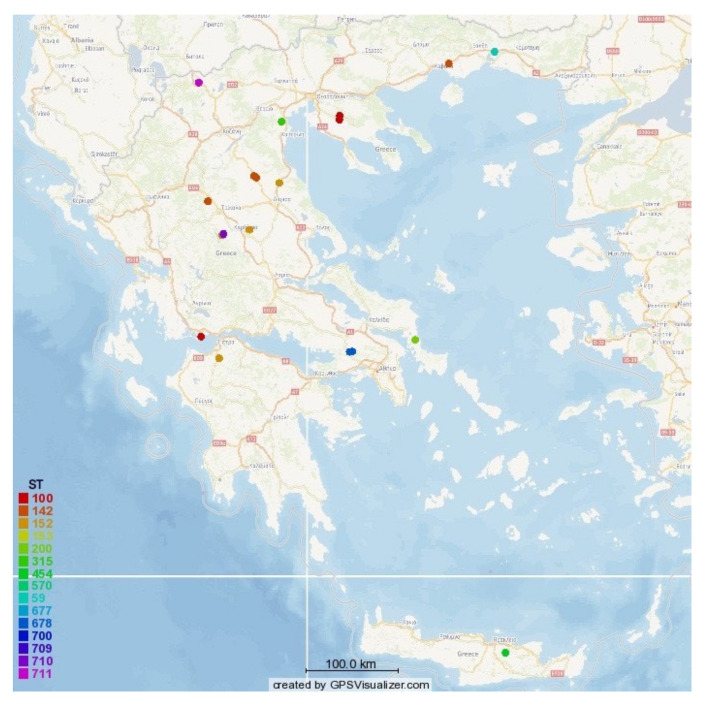
Map of Greece indicating the location of sheep farms where 33 *S. epidermidis* isolates from cases of ovine mastitis were recovered, according to the ST of the isolates (drawn by using the GPS-visualiser). ST: sequence type; GPS: Global Positioning System.

**Figure 7:**
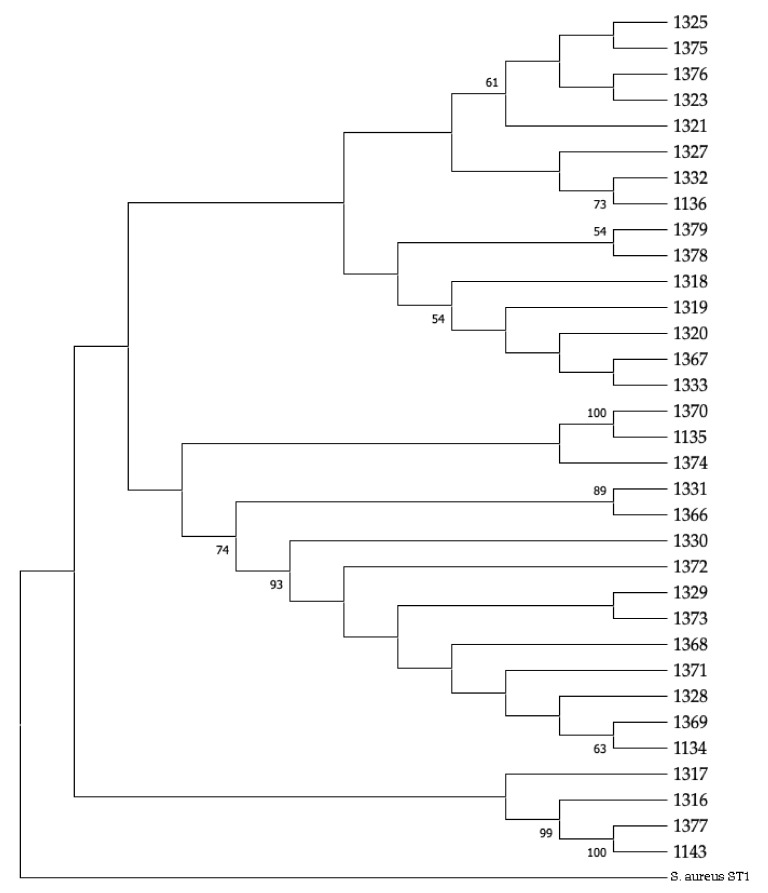
A maximum likelihood phylogenetic tree of 33 *S. epidermidis* isolates from cases of ovine mastitis in Greece in the MLST database and *S. aureus* ST1 as an outgroup. Bootstrap values of >50 are shown in the graph, and numbers to the right of the tree refer to the reference ID in the MLST database, which were used in the analysis. MLST: multi-locus sequence typing; ST: sequence type; ID: isolate identity in the database.

The spanning tree of all the isolates recovered from cases of ovine mastitis in Greece is shown in Figure 5. The tree includes 15 nodes and 14 edges (i.e., branches) (median length of edges: 2). The location of the farms from which isolates originated is shown in Figure 6. The phylogenetic tree of the isolates is shown in Figure 7.

There was evidence of a difference in the frequency of alleles of the genes *aroE* (*p* = 0.016) and *tpiA* (*p* = 0.004) between isolates from mastitis or from mammary carriage (Table 3) but not in the frequency of alleles of the other five genes (*p* > 0.10). There was also evidence of a difference in the frequency of alleles of the genes *arcC* (*p* < 0.0001), *pyrR* (*p* = 0.0004) and *tpiA* (*p* = 0.031) between isolates from mastitis or from humans in Greece (Table 4).

Of the 15 STs in which the above isolates were distributed, in 6 (40.0%), there were also isolates of human origin. These STs were ST59, ST142, ST152, ST200, ST225 and ST315. None of the human origin isolates in these STs was recovered in Greece.

#### 3.2.2. Resistance to Antimicrobial Agents

Of the 33 isolates, 19 (57.6%) were found to be resistant to at least one antimicrobial agent and 2 (6.1%) were found to be multi-drug resistant. Specifically, 16 isolates (48.5%) were resistant to tetracycline (TE), 11 (33.3%) to penicillin and ampicillin (P/AMP), 4 to fucidic adic, 3 to erythromycin, 1 to clindamycin and 1 to cefoxitin. The resistance profiles of the isolates are in Table S5.

The following resistance-related genes were identified in the isolates: *tetK* (15 isolates), *tetT* (5 isolates), *msrA* (2 isolates), *tetM* (2 isolates), *tetS* (1 isolate) and *ermC* (1 isolate).

**Figure 8:**
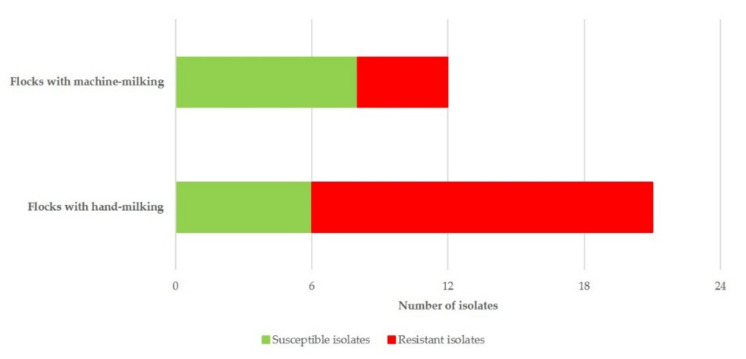
Frequency of recovery of *S. epidermidis* isolates from cases of ovine mastitis in Greece (*n* = 33) in the MLST database according to antimicrobial agent susceptibility/resistance of the isolates and milking mode applied in the flocks. MLST: multi-locus sequence typing.

In multivariable analysis, only hand-milking applied in a flock emerged to be significant (*p* = 0.026) for the presence of resistance to antimicrobial agents in the isolates (Table 5 and Figure 8).

#### 3.2.3. Associations between MLST and Resistance to Antimicrobial Agents

There was no association between the phenotypic resistance to any of the above antimicrobial agents or the presence of any of the various resistance genes therein and the ST of the isolates (*p* > 0.24 for all comparisons); in this respect, the presence of susceptible and resistant isolates within the same ST was also noted. In addition, there was no association between the presence of the various resistance genes in the isolates and the alleles in each of the seven genes (*p* > 0.11 in all cases).

The isolates from flocks in which machine-milking was applied were distributed in 8 STs, and those from flocks in which hand-milking was applied were distributed in 10 STs; there was no difference in the frequency of isolates from either type of flocks among various STs (*p* = 0.29).

There was a significant difference in the frequency of the various STs between isolates from cattle farms and sheep flocks in which hand-milking was applied (*p* = 0.018) but not in the frequency of the various STs between isolates from cattle and sheep flocks in which machine-milking was applied (*p* = 0.11) (Table S6).

Finally, there was no difference in the proportion of STs between isolates from flocks with machine- or hand-milking in which there were also isolates of human origin (in the entirety of the MLST database) (62.5% versus 30.0%, *p* = 0.17) (Table S7).

## 4. Discussion

In the study of pathogen biology, the genetic diversity and spatiotemporal spread of bacteria can be useful to shed light on the origin and epidemic history of pathogens, from reservoir dynamics to the emergence and adaptation to new hosts, and their diversity [42]. This can be of importance for understanding the dissemination of strains between hosts of the same or different species, as well as in the control of microbial diseases.

By using the MLST-based classification, DNA-level differences of the bacteria under study can be taken into account. The data generated are deposited in an easily accessed database and thus can be shared among many laboratories throughout the world [43]. The MLST scheme was selected over older typing methods, e.g., pulsed-field gel electrophoresis, because objective results were exported, and these could be comparable worldwide. However, as inclusion of isolates into the database is not compulsory, the database is not exhaustive, but is limited only to isolates for which detailed identification and typing were performed, followed by the specific inclusion into the database.

With 949 STs in the database, the genetic diversity of *S. epidermidis* becomes apparent. The recovery of isolates from 46 countries in the world also indicates the significant geographic dissemination. Isolates were recovered mostly from people.

The findings confirm that in animals, *S. epidermidis* is primarily a mammary pathogen. In the past, it was suggested that *S. epidermidis* isolates causing mammary infections may be of human origin [44], a view reiterated by Thorberg et al. [45] more recently. The present results do not align with the above hypothesis, as only a proportion (27.1%) of STs with isolates of mastitis origin also included isolates of human origin. Certainly, one may consider that isolates from animal mastitis had undergone changes to better adapt within the environment of the mammary gland. It is, however, noteworthy that recent studies have now confirmed *S. epidermidis* as a causal agent of mastitis in women [46,47], although no such isolates have been included in the database.

Using the Grape-Tree visualisation of genetic clusters, we found groups of isolates with identical STs depicted in single nodes (the diameter of which reflected the number of isolates). This analysis contributed to further investigation of the association of *S. epidermidis* isolates according to ST and geographic origin.

Genetic diversity among isolates recovered from bovine or ovine mastitis was found. Then, a lack of differences between isolates of bovine origin (i.e., from farms where, most probably, machine-milking was applied) and isolates from flocks with machine-milking, coupled with contrasting differences between the former isolates and ones from flocks with hand-milking, also became evident. A hypothesis could be that, possibly, the use of the milking system might lead to some bacterial adaptation to the relevant environments, as seen with the difference in STs in which respective isolates were distributed.

The 33 isolates recovered in Greece were genotyped using the MLST method, and the concatenated sequences of the seven housekeeping genes were used to infer their phylogenetic relationships. These isolates were assigned to 15 different STs, which was indicative of a high genetic diversity, given also that the median distance between STs was two alleles. Miragaia et al. [48] characterised 217 isolates of *S. epidermidis* and identified 74 different STs, while Sharma et al. [49] detected 44 unique STs in 100 isolates of *S. epidermidis*. Recombination events and selective pressures (possibly antibiotic usage) seem to shape the genetic diversity of the species.

According to phylogenetic analysis, the haplotypes were organised in three groups (Figure 7), with the group in proximity to *S. aureus* used as the outgroup, including three STs, while the other groups included six STs each. However, the topology of the tree did not reveal any phylogeographic signal, since the grouping did not correspond to the geographic origin of the isolates.

It is noteworthy that the imputation method used to identify the missing STs was in concordance with the results of Grape-Tree (Figure 5), which grouped the imputed isolates (before the imputation) along with the isolates, which were successfully genotyped in all seven loci and were assigned to an ST. Imputation is a widely used method for genotype prediction, especially in species with well-known haplotypes, and in the present isolates, the MLST database allowed this opportunity [50]. In any case, network analysis was performed without imputing the missing values and the results are in accord with the imputed values.

These 33 isolates were recovered from two extensive and lengthy field studies on ovine mastitis. There appeared to be a wide dissemination of *S. epidermidis* across the country, from northern Greece to the island of Crete. The evaluation of the location of the farms, where these isolates were recovered, also indicated that isolates included in the same ST were recovered in geographically distant locations. Animal movements through sales or seasonal transhumance of flocks might have contributed, as animals of different flocks would mix in the process. The countrywide recovery of isolates resistant to antimicrobial agents lends support to this hypothesis.

The frequency of resistance to antimicrobial agents was higher than that reported elsewhere. For example, Onni et al. [51], in Italy, found that the resistance rate specifically in *S. epidermidis* isolates from mastitis was 38%. This is in line with the increased use of antimicrobial agents in Greece, associated with the consequential development of resistance to these agents [52,53].

Among the various genes associated with resistance to antimicrobial agents, those of the *tet* operon predominated, a finding which is in line with previous relevant work performed in staphylococci from milk of cows [54].

In general, hand-milking has been associated with a higher frequency of mammary infections in ewes [55,56]; it has been documented that staphylococci can be transmitted from milkers’ hands to the teat of ewes, subsequently entering into the mammary gland and causing mastitis [57,58]. Hence, there would be a higher need for administration of antimicrobial agents to the animals for treatment of the infection [59], thus increasing the risk of the causal bacteria developing resistance, as found in the present study. In contrast, in flocks in which machine-milking is applied, other means of control (e.g., post-milking teat disinfection, thorough cleaning of milking parlours) would usually be used, which could contribute to limiting the bacterial burden and preventing infections. In any case, the number of the isolates recovered should be considered.

It is noteworthy that no isolates recovered in Greece from sheep or people assigned into the same ST have been reported, despite the interactions, as described above. We postulate that farmers, who live mainly in rural areas, would not visit physicians for treatment, unless an infection was severe; they would also visit local physicians, with no need to referral to a hospital, where detailed sampling, bacterial isolation, MLST and reporting into the database would be feasible.

## 5. Conclusions

The evaluation of the MLST database indicates a genetic and geographic diversity of *S. epidermidis* isolates. In animals, *S. epidermidis* is primarily a mammary pathogen. Genetic diversity among isolates from bovine or ovine mastitis was found.

In isolates recovered in Greece from cases of ovine mastitis, there was an increased frequency of resistance mainly to penicillin/ampicillin and tetracycline. There was association between the presence of resistance and hand-milking in the respective flocks. No association was found between the presence of resistance and MLST of the isolates.

## Figures and Tables

**Table 1:** Details of alleles detected across all 7 genes evaluated in *S. epidermidis* isolates in the MLST database (*n* = 1593).

Alleles	*arcC*	*aroE*	*gtr*	*mutS*	*pyrR*	*tpiA*	*yqiL*
Total no. of alleles detected	72	67	79	43	58	63	68
Most frequent allele	1	1	2	2	2	1	1
Isolates (n)	623	994	612	596	511	939	758
Second-most frequent allele	7	2	1	1	4	16	4
Isolates (n)	223	104	312	302	277	139	101

MLST: multi-locus sequence typing.

**Table 2:** Frequency of alleles of the genes *arcC* and *yqiL* in *S. epidermidis* isolates from cases of mastitis in the MLST database, recovered from cattle (*n* = 50) or sheep (*n* = 33).

arcC			yqiL		
Allele	Isolates from Bovine Mastitis (n)	Isolates from Ovine Mastitis (n)	Allele	Isolates from Bovine Mastitis (n)	Isolates from Ovine Mastitis (n)
1	33	9	1	21	29
2	8	18	3	0	1
7	3	0	4	3	0
8	0	3	7	7	0
12	4	1	8	3	1
19	1	0	10	7	0
28	1	0	11	0	2
65	0	2	18	6	0
			19	1	0
			21	1	0
			49	1	0

MLST: multi-locus sequence typing.

**Table 3:** Frequency of alleles of the genes *aroE* and *tpiA* in *S. epidermidis* isolates from cases of ovine mastitis (*n* = 33) or ovine mammary carriage (*n* = 4) in the MLST database, recovered in Greece.

aroE			tpiA		
Allele	Isolates from Ovine Mastitis (n)	Isolates from Mammary Carriage (n)	Allele	Isolates from Ovine Mastitis (n)	Isolates from Mammary Carriage (n)
1	25	2	1	16	3
2	1	0	5	1	0
3	0	1	16	2	0
13	0	1	19	0	1
25	1	0	48	1	0
51	1	0	2		
59	1	0			

MLST: multi-locus sequence typing.

**Table 4:** Frequency of alleles of the genes *arcC*, *pyrR* and *tpiA* in *S. epidermidis* isolates from cases of ovine mastitis (*n* = 33) or human patients (*n* = 10) in the MLST database, recovered in Greece.

arc			pyrR			tpiA		
Allele	Isolates from Ovine Mastitis (n)	Isolates of Human Origin (n)	Allele	Isolates from Ovine Mastitis (n)	Isolates of Human Origin (n)	Allele	Isolates from Ovine Mastitis (n)	Isolates of Human Origin (n)
1	9	2	1	0	1	1	17	5
2	18	0	2	18	1	3	0	1
3	0	1	3	1	2	4	0	1
7	0	6	4	0	5	5	1	0
8	3	0	6	1	0	7	0	2
12	1	0	8	2	0	13	0	1
22	0	1	9	2	0	16	4	0
65	2	0	10	0	1	48	2	0
			20	9	0			

MLST: multi-locus sequence typing.

**Table 5:** Results of multivariable analysis for the presence of resistance to antimicrobial agents in *S. epidermidis* isolates from cases of subclinical mastitis in sheep in Greece.

Milking Techniques Applied in the Flock	Proportion of Flocks in Which Resistant *S. epidermidis* Were Recovered	Odds Ratio ^1^ (95%CI ^2^)	*p*
Machine-milking (*n* = 12)	33.3%	reference	
Hand-milking (*n* = 21)	71.4%	5.000 (1.084–23.061)	0.039

^1^ Odds ratios calculated against the lowest frequency of the presence of resistance; ^2^ CI: confidence interval.

## Data Availability

Data presented in this study are available (a) in the open source database www.pubmlst.org (accessed on 10 November 2020) and (b) in the main text and the supplementary materials of the paper.

## Supplementary Materials

The following are available online at https://www.mdpi.com/2079-7737/10/3/170/s1: Table S1: Primers used for detection of genes for inclusion in the MLST database in 33 *S. epidermidis* isolates recovered from subclinical mastitis in ewes in Greece (MLST: multi-locus sequence typing); Table S2: Primers used and work conditions undertaken for detection of resistance genes in *S. epidermidis* isolates recovered from subclinical mastitis in ewes in Greece; Table S3: Husbandry factors evaluated for potential association with antimicrobial resistance of 33 *S. epidermidis* isolates recovered from subclinical mastitis in ewes in Greece; Table S4: Identity and details in the MLST database of 33 *S. epidermidis* isolates recovered from subclinical mastitis in ewes in Greece (MLST: multi-locus sequence typing); Table S5: Profiles of antimicrobial resistance of *S. epidermidis* isolates recovered from subclinical mastitis in ewes in Greece; Table S6: Frequency of *S. epidermidis* isolates from cattle farms and of isolates from sheep flocks, machine- or hand-milked, in Greece, according to STs in the MLST database (MLST: multi-locus sequence typing; ST: sequence type); Table S7: STs with *S. epidermidis* isolates from sheep flocks, machine- or hand-milked, in Greece, with simultaneous inclusion of isolates from human sources in the MLST database (MLST: multi-locus sequence typing; ST: sequence type).
