# Supplementary material for: MLST-Based Analysis and Antimicrobial Resistance of Staphylococcus epidermidis from Cases of Sheep Mastitis in Greece
Source: Biology (Basel). 2021 Feb 24;10(3):170. doi: 10.3390/biology10030170 (PMC7996216; doi:10.3390/biology10030170)
Supplement: Supplementary file 1 [file biology-10-00170-s001.pdf]

MLST-Based Analysis and Resistance to Antimicrobial Agents of *Staphylococcus epidermidis* from Cases of Sheep Mastitis in Greece

Eleni I. Katsarou, Dimitrios C. Chatzopoulos, Themis Giannoulis, Katerina S. Ioannidi, Angeliki I. Katsafadou, Panagiota I. Kontou, Daphne S. Lianou, Zissis Mamuris, Vasia S. Mavrogianni, Charalambia K. Michael, Elias Papadopoulos, Efthimia Petinaki, Styliani Sarrou, Natalia G.C. Vasileiou, George C. Fthenakis

Table S1. Primers used for detection of genes for inclusion in the MLST database, in 33 *S. epidermidis* isolates recovered from subclinical mastitis in ewes in Greece (MLST: Multi Locus Sequence Typing).

Gene	Primers	Sequences	Product size (bp)
arcC (carbamate kinase)	arcC-F	TGTGATGAGCACGCTACCGTTAG	465
	arcC-R	TCCAAGTAAACCCATCGGTCTG	
aroE (shikimate dehydrogenase)	aroE-F	CATTGGATTACCTCTTTGTTCAGC	420
	aroE-R	CAAGCGAAATCTGTTGGGG	
gtr (ABC transporter)	gtr-F	CAGCCAATTCTTTTATGACTTTT	438
	gtr-R	GTGATTAAAGGTATTGATTTGAAT	
mutS (DNA mismatch repair protein)	mutS-F3	GATATAAGAATAAGGGTTGTGAA	412
	mutS-R3	GTAATCGTCTCAGTTATCATGTT	
pyrR (pyrimidine operon regulatory protein)	pyr-F2	GTTACTAATACTTTTGCTGTGTTT	428
	pyr-R4	GTAGAATGTAAAGAGACTAAAATGAA	
tpiA (triosephosphate isomerase)	tpi-F2	ATCCAATTAGACGCTTTAGTAAC	424
	tpi-R2	TTAATGATGCGCCACCTACA	
*yqi*L (acetyl coenzyme A acetyltransferase)	yqiL-F2	CACGCATAGTATTAGCTGAAG	416
	yqiL-R2	CTAATGCCTTCATCTTGAGAAATAA	

Table S2. Primers used and work conditions undertaken for detection of resistance genes in 33 *S. epidermidis* isolates recovered from subclinical mastitis in ewes in Greece.

Gene	Primer sequence	Concentration (μM)	Product size (bp)	Annealing temperature (°C)	Reference
ermA	Fw-TCTAAAAAGCATGTAAAAGAA<br>Rv-CTTCGATAGTTTATTAATATTAGT	0.2	645	55	[1]
ermB	Fw-GAAAAGGATCTCAACCAAATA<br>Rv-AGTAACGGTACTTAAATTGTTTAC	0.2	639	55	[1]
ermC	Fw-TCAAAACATAATATAGATAAA<br>Rv-GCTAATATTGTTTAAATCGTCAAT	0.2	649	52	[1]
ermT	Fw-GGTGTAATTATGTAACCGCCA<br>Rv-ACTTCCTGTAGCTGTGCTTTC	0.2	218	52	[1]
ermY	Fw-ATTACTTCGAAACGTAATATAGAT<br>Rv-ATAGCTATTGAAAAGAGACAAGA	0.2	~ 700	60	[2]
lnuA	Fw-GGTGGCTGGGGGGTAGATGTATTAACTGG<br>Rv-GCTTCTTTTGAAATACATGGTATTTTTCGA	0.2	323	55	[3]
lnuB	Fw-CCTACCTATTGTTTGTGGAA<br>Rv-ATAACGTTACTCTCCTATTC	0.2	944	50	[4]
lnuC	Fw-GTAGATGCTCTTCTTGGAT<br>Rv-TTCTACCGGAAAACAATCC	0.2	246	55	[5]
lsaA	Fw-GATCAGGCAAATATCACGATG<br>Rv-CTTCATGAAATGTGCATCATGC	0.2	~ 1400	60	[6]
mphC	Fw-ATCTCATTGAATGAATCAGGAC<br>Rv-CTACTCTTCCATACCTAACTC	0.2	~ 850	60	[2]
msrA	Fw-GCAAATGGTGTAGGTAAGACAACT<br>Rv- ATCATGTGATGTAAACAAAAT	0.2	399	55	[1]
tetK	Fw-TATTTTGGCTTTGTATTCTTTCAT<br>Rv-GCTATACCTGTTCCCTCTGATAA	0.2	1159	59	[7]
tetL	Fw-ATAAATTGTTTCGGGTCGGTAAT<br>Rv-AACCAGCCAACTAATGACAATG	0.2	1077	59	[7]
tetM	Fw-GAACTCGAACAAGAGGAAAGC<br>Rv-ATGGAAGCCCAGAAAGGAT	0.2	740	54	[8]
tetS	Fw-ATCAAGATATTAAGGAC<br>Rv-TTCTCTATGTGGTAATC	0.2	573	55	[9]

tetT	Fw-CAGTGCGAATATAAGGACACGTC<br>Rv-CAAGCCTTCTCTACAGCATC	0.2	644	61	[10]
vgaA	Fw-CTTGTCTCCTCCGCGAATAC<br>Rv-AGTGGTGGTGAAGTAACACG	0.2	659	55	[11]

References

1. Alós, J.I.; Aracil, B.; Oteo, J.; Torres, C.; Gómez-Garcés, J.L.; Spanish Group for the Study of Infection in the Primary Health Care Setting. High prevalence of erythromycin-resistant, clindamycin/miocamycin-susceptible (M phenotype) *Streptococcus pyogenes*: results of a Spanish multicentre study in 1998. *J. Antimicrob. Chemother.* **2000**, *45*, 605–609.
2. Matsuoka, M.; Inoue, M.; Endo, Y.; Nakajima, Y. Characteristic expression of three genes, msr(A), mph(C) and erm(Y), that confer resistance to macrolide antibiotics on *Staphylococcus aureus*. *FEMS Microbiol. Lett.* **2003**, *220*, 287–293.
3. Lina, G.; Quaglia, A.; Reverdy, M.E.; Leclercq, R.; Vandenesch, F.; Etienne, J. Distribution of genes encoding resistance to macrolides, lincosamides, and streptogramins among staphylococci. *Antimicrob. Agents Chemother.* **1999**, *43*, 1062–1066.
4. Bozdogan, B.; Berrezouga, L.; Kuo, M.S.; Yurek, D.A.; Farley, K.A.; Stockman, B.J.; Leclercq, R. A new resistance gene, *linB*, conferring resistance to lincosamides by nucleotidylation in *Enterococcus faecium* HM1025. *Antimicrob. Agents Chemother.* **1999**, *43*, 925–929.
5. Gravey, F.; Galopin, S.; Grall, N.; Auzou, M.; Andremont, A.; Leclercq, R.; Cattoir, V. Lincosamide resistance mediated by lnu(C) (L phenotype) in a *Streptococcus anginosus* clinical isolate. *J. Antimicrob. Chemother.* **2013**, *68*, 2464–2467.
6. Malbruny, B., Werno, A.M.; Murdoch, D.R.; Leclercq, R.; Cattoir, V. Cross-resistance to lincosamides, streptogramins A, and pleuromutilins due to the lsa(C) gene in *Streptococcus agalactiae* UCN70. *Antimicrob. Agents Chemother.* **2011**, *55*, 1470–1474.
7. Trzcinski, K.; Cooper, B.S.; Hryniewicz, W.; Dowson, C.G. 2000. Expression of resistance to tetracyclines in strains of methicillin-resistant Staphylococcus aureus. *J. Antimicrob. Chemother.* **2000**, *45*, 763–770.
8. Olsvik, B.; Olsen, I.; Tenover, F.C. Detection of tet(M) and tet(O) using the polymerase chain reaction in bacteria isolated from patients with periodontal disease. *Oral Microbiol. Immunol.* **1995**, *10*, 87-92.
9. Gevers, D.; Huys, G.; Swings, J. *In vitro* conjugal transfer of tetracycline resistance from Lactobacillus isolates to other Gram-positive bacteria. *FEMS Microbiol. Lett.* **2003**, *225*, 125–130.
10. Clermont, D.; Chesneau, O.; De Cespédès, G.; Horaud, T. New tetracycline resistance determinants coding for ribosomal protection in streptococci and nucleotide sequence of *tet(T)* isolated from *Streptococcus pyogenes* A498. *Antimicrob. Agents Chemother.* **1997**, *41*, 112–116.
11. Soltani, M.; Beighton, D.; Philpott-Howard, J.; Woodford, N. Mechanisms of resistance to quinupristin-dalfopristin among isolates of *Enterococcus faecium* from animals, raw meat, and hospital patients in Western Europe. *Antimicrob. Agents Chemother.* **2000**, *44*, 433–436.

Table S3. Husbandry factors applied in sheep flocks, which were evaluated for potential association with resistance to antimicrobial agents of 33 *S. epidermidis* isolates recovered from subclinical mastitis in ewes in Greece.

Management system applied in the flock (description according to EFSA classification) [1}
Stage of the lactation period at sampling (month)
Milking technique applied in the flock (machine milking, hand-milking)
Application of post-milking teat dipping (yes, no)
Intramammary administration of antimicrobial agents at the end of the lactation period (yes, no)
Vaccination against staphylococcal mastitis (yes, no)

References

1. European Food Safety Authority. Scientific opinion on the welfare risks related to the farming of sheep for wool, meat and milk production. *EFSA J.* **2014**, *12*, 3933-4060.

Table S4. Identity and details in the MLST database of 33 *S. epidermidis* isolates recovered from subclinical mastitis in ewes in Greece (MLST: Multi Locus Sequence Typing).

Isolate ID	Alleles of relevant genes							ST
	arcC	*aroE*	*gtr*	*mutS*	*pyrR*	*tpiA*	*yqiL*	
1134	2	1	1	17	20	1	1	709
1135	8	51	56	37	9	16	1	710
1136	1	1	57	6	2	16	7	711
1143	65	59	5	5	8	48	11	700
1316	12	25	9	8	6	5	8	315
1317	1	1	2	2	3	1	3	200
1318	2	1	2	2	2	1	1	142
1319	2	1	2	2	2	1	1	142
1320	2	1	2	2	2	1	1	142
1321	1	1	2	6	2	1	1	152
1323	1	1	2	6	2	1	1	152
1325	1	1	2	6	2	1	1	152
1327	1	1	2	1	2	1	8	454 [1]
1328	2	1	1	2	20	1	1	100
1329	2	1	1	2	20	1	1	100
1330	2	1	1	2	20	1	1	100
1331	2	1	1	1	2	1	1	59
1332	1	1	2	6	2	16	7	570
1333	2	1	2	2	2	2	1	142
1366	8	2	1	1	2	1	1	677
1367	2	1	2	2	2	1	1	142
1368	2	1	1	2	20	1	1	100
1369	2	1	1	17	20	1	1	709
1370	8	51	56	37	9	16	1	710
1371	2	1	1	2	20	1	1	100
1372	2	1	1	2	20	1	1	100
1373	2	1	1	2	20	1	1	100
1374	2	1	6	2	2	2	1	153
1375	1	1	2	6	2	1	1	152
1376	1	1	2	6	2	1	1	152
1377	65	59	5	5	8	48	11	700
1378	2	1	2	2	2	1	7	678
1379	2	1	2	2	2	1	7	678

[1] Red lettering indicates missing alleles (completed by means of an imputational method) and missing STs (thereafter completed as the most probable ST).

Table S5. Profiles of resistance to antimicrobial agents of 33 *S. epidermidis* isolates recovered from subclinical mastitis in ewes in Greece.

Isolate identity in the MLST database [1]	Antimicrobial agents to which resistance was recorded [2]
1134	P, AMP
1135	TE, FA
1136	none
1143	E, TE
1316	none
1317	P, AMP, TE
1318	P, AMP, FA
1319	P, AMP, TE
1320	P, AMP, TE
1321	P, AMP, TE
1323	P, AMP, **TE**
1325	none
1327	none
1328	none
1329	none
1330	none
1331	E, CC,TE, FOX
1332	none
1333	none
1366	P, AMP, TE, FA
1367	P, AMP, TE
1368	P, AMP, TE
1369	P, AMP
1370	TE, FA
1371	TE
1372	none
1373	TE
1374	none
1375	none
1376	none
1377	E, TE
1378	TE
1379	none

[1] MLST: Multi Locus Sequence Typing, [2] AMP: ampicillin, CC: clindamycin, E: erythromycin, FA: fucidic acid, FOX: cefoxitin, P: penicillin, TE: tetracycline.

Table S6. Frequency of *S. epidermidis* isolates from cattle farms and of isolates from sheep flocks, machine-or hand-milked, in Greece, according to STs in the MLST database (MLST: Multi Locus Sequence Typing, ST: sequence type).

Isolates from cattle farms [1]		Isolates from sheep flocks with machine-milking		Isolates from sheep flocks with hand-milking	
ST (n=36)	no. of isolates (n=50)	ST (n=8)	no. of isolates (n=12)	ST (n=10)	no. of isolates (n=21)
5	2	59	1	100	5
7	1	100	2	142	2
48	2	142	3	152	3
54	1	152	2	153	1
57	1	200	1	454	1
59	5	315	1	677	1
91	6	570	1	678	2
92	1	711	1	700	2
93	1			709	2
94	2			710	2
98	1				
99	1				
100	1				
101	1				
102	1				
103	1				
104	1				
105	1				
106	1				
107	1				
108	1				
109	2				
110	1				
111	2				
112	1				
114	1				
329	1				
441	1				
443	1				
444	1				
452	1				
453	1				
454	1				
473	1				
474	1				
639	1				

1. Details obtained from the MLST-database.

Table S7. STs with *S. epidermidis* isolates from sheep flocks, machine-or hand-milked, in Greece, with simultaneous inclusion of isolates from human sources in the MLST database (MLST: Multi Locus Sequence Typing, ST: sequence type).

STs with isolates from sheep flocks with machine-milking (n=8)	STs with isolates from sheep flocks with hand-milking (n=10)	Inclusion of isolates from human sources in the ST
59		yes
100	100	no
142	142	yes
152	152	yes
	153	yes
200		yes
315		yes
	454	no
570		no
	677	no
	678	no
	700	no
	709	no
	710	no
711		no

© 2020 by the authors. Licensee MDPI, Basel, Switzerland. This article is an open access article distributed under the terms and conditions of the Creative Commons Attribution (CC BY) license (http://creativecommons.org/licenses/by/4.0/).
